# A simple, non‐toxic method for separating seeds based on density, and its application in isolating *Arabidopsis thaliana* seed oil mutants

**DOI:** 10.1002/aps3.11332

**Published:** 2020-04-20

**Authors:** Gillian H. Dean, Flora Pang, George W. Haughn, Ljerka Kunst

**Affiliations:** ^1^ Department of Botany University of British Columbia 6270 University Boulevard Vancouver V6T 1Z4 Canada

**Keywords:** *Arabidopsis thaliana*, seed density, seed oil, seed separation

## Abstract

**Premise:**

Seed oil is an economically important trait in *Brassica* oilseed crops. A novel method was developed to isolate *Arabidopsis thaliana* seeds with altered oil content.

**Methods and Results:**

In *A. thaliana*, seed oil content is correlated with seed density, with high‐oil seeds being less dense than wild type and tending to float in solution, and low‐oil seeds being denser and tending to sink. In contrast to previous methods, which used toxic chemicals and density gradient centrifugation, different concentrations of calcium chloride (CaCl_2_) were employed to separate seeds without the need for centrifugation. The method was validated using known seed oil mutants, and 120,822 T‐DNA mutagenized *A. thaliana* lines were then screened for novel seed density phenotypes.

**Conclusions:**

A number of candidate mutants, as well as new alleles of two genes known to influence seed oil biosynthesis, were successfully isolated.

The control and manipulation of seed oil content has been the subject of much research, especially in *Brassica* L. oilseed crops. One approach to identifying new candidate genes involved in this economically important trait is to identify mutants with either increased or decreased seed oil content from large populations of mutagenized plants in the model oilseed plant *Arabidopsis thaliana* (L.) Heynh. A successful forward genetic screen is dependent on a robust, simple, and time‐efficient screening protocol.

In *A. thaliana*, seed oil content is correlated with seed density. Seeds with high oil content are less dense than wild type, and seeds with low oil content are denser than wild type. This characteristic has been successfully exploited in previous studies to screen mutant populations of *A. thaliana* for seeds with altered oil content. Such screens used density gradient centrifugation, with gradients prepared using mixtures of 1‐bromohexane and 1,6‐dibromohexane (Focks and Benning, 1998) or 1‐bromohexane, 1,6‐dibromohexane, and mineral oil (Shen et al., 2006). However, these chemicals are harmful and flammable, require special handling, and are difficult to dispose. Therefore, we developed an alternative protocol that is safer and faster because it uses calcium chloride (CaCl_2_) instead of bromohexane and does not require centrifugation.

The use of CaCl_2_ to separate soybean seeds from those of several weed species has been described previously (Johnston et al., 1978). Here, we present a significantly modified protocol that allows separation of seeds within the same species and on a scale suitable for the numbers of seeds recovered from *A. thaliana* mutagenesis, and demonstrate that novel mutants can be isolated from mutagenized populations using this method.

## METHODS AND RESULTS

### Inhibition of mucilage extrusion using ethanol

**Figure 1:**
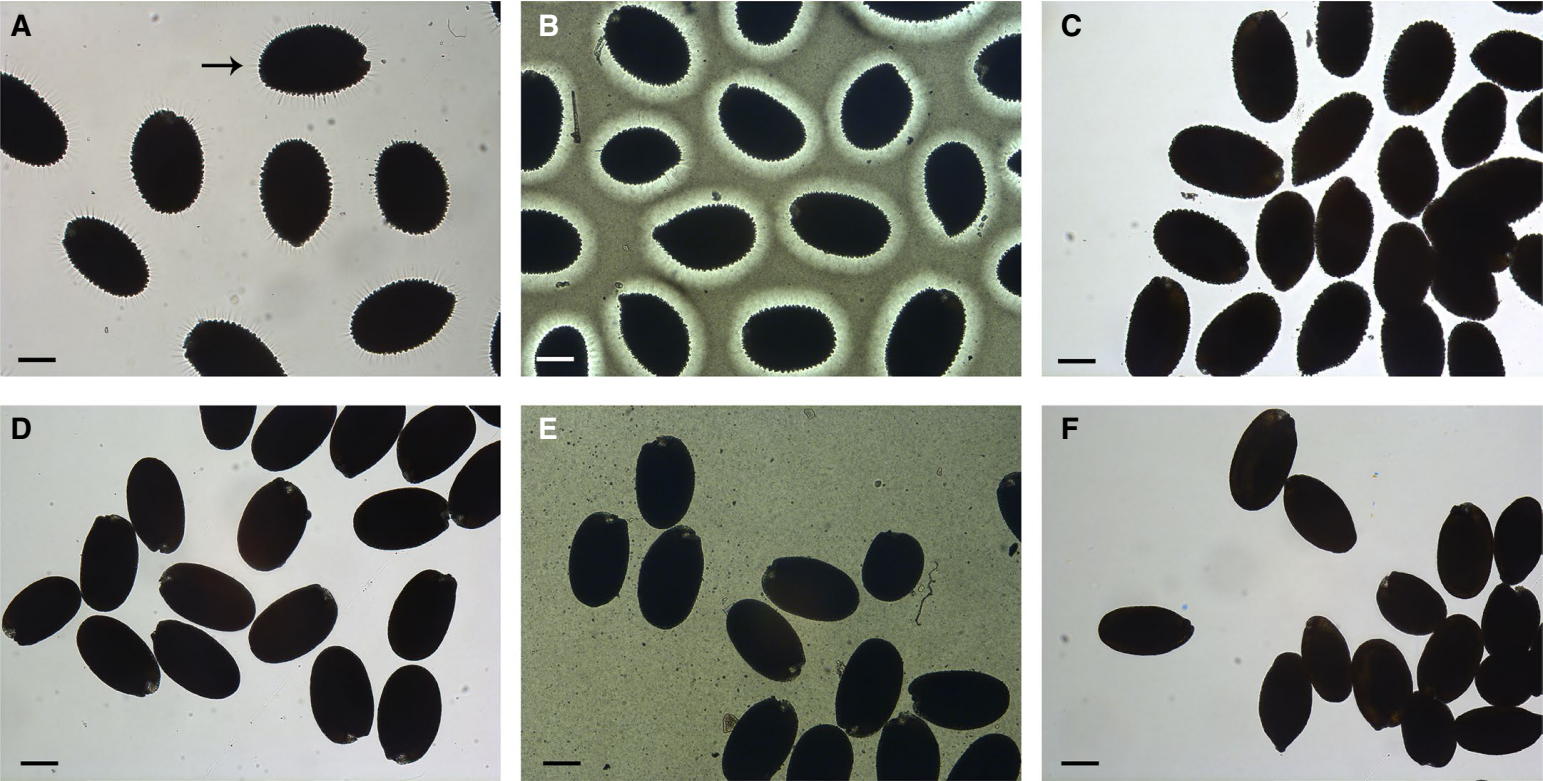
Inhibition of seed mucilage extrusion using ethanol. Mature wild‐type Col‐0 (A–C) and *mum2* (D–F) *Arabidopsis thaliana* seeds were hydrated in water (A, D), water with India ink (B, E), or 30% (v/v) ethanol (C, F). Mucilage is released from Col‐0 in water (A, arrow indicates ray‐like structures in extruded mucilage) and in water with India ink added (B), where the presence of the mucilage halo is highlighted because India ink cannot penetrate the mucilage, but mucilage is not released in 30% (v/v) ethanol (C). *mum2* does not release mucilage in water (D; E, the addition of India ink confirms that no mucilage halo is extruded) or 30% (v/v) ethanol (F). Scale bar = 150 μm.

The capacity of *A. thaliana* seeds to float or sink in water is affected by the presence of seed mucilage as well as their seed oil content. Mature *A. thaliana* seeds release a large amount of pectinaceous, hydrophilic mucilage on contact with water (Beeckman et al., 2000; Western et al., 2000; Windsor et al., 2000). In contrast, *A. thaliana* mutants such as *mum2* (Dean et al., 2007; Macquet et al., 2007) that do not release mucilage tend to be difficult to wet and will float on the surface of water. In addition, if *A. thaliana* plants are not watered until all of the seeds have completed development, mucilage release is impaired. In order to ensure that differences in mucilage extrusion do not influence the seed density screen, ethanol was included in the CaCl_2_ solutions used for density screening. Wild‐type *A. thaliana* seeds (Col‐0 ecotype) were utilized to determine the minimum concentration of ethanol that would completely inhibit mucilage release, and *mum2* was included to demonstrate that ethanol aids wetting of seeds that do not release mucilage. Approximately 30 mature *A. thaliana* seeds were placed into 1.5‐mL microcentrifuge tubes, and 1 mL of water or solutions of 10%, 20%, 30%, or 40% (v/v) ethanol in water was added. Tubes were vortexed briefly to ensure all seeds were immersed in the solution and then left to settle for 30 min. The seeds were transferred to a glass dimple slide and viewed under a compound microscope. The wetting of *mum2* seeds was improved in all concentrations of ethanol. When wild‐type Col‐0 seeds were hydrated in water, they released a large halo of mucilage, which was observed as rays extending from the seed (Fig. 1A, arrow). In addition, the presence of the mucilage resulted in noticeable spaces between adjacent hydrated seeds. In contrast, *mum2* seeds do not release mucilage in water; rays were not visible and the seeds were close together (Fig. 1D). The presence or absence of mucilage was more easily observed by the addition of India ink, which cannot penetrate the mucilage halo (Fig. 1B, E). Wild‐type seeds released mucilage in 10% and 20% ethanol, but not in 30% (Fig. 1C) or 40% ethanol; no rays were visible and the seeds were in close proximity. Hydration of *mum2* in 30% ethanol (Fig. 1F) gives similar results to hydration in water (Fig. 1D).

### *Arabidopsis thaliana* oil mutants used for method development

The feasibility of the approach was demonstrated using previously characterized *A. thaliana* mutants with altered seed oil content. Two mutants, *mum4/rhm2* (Western et al., 2001, 2004; Usadel et al., 2004) and *gl2* (Rerie et al., 1994; Western et al., 2004), are known to have high seed oil content and lack seed mucilage (Shen et al., 2006; Shi et al., 2012). Because two different seed characteristics were altered in these mutants, the *mum2* mutant, which fails to extrude mucilage (Dean et al., 2007; Macquet et al., 2007) but is expected to have seed oil levels similar to wild type, was also included as a control. The low‐seed‐oil mutants *fus3* (Keith et al., 1994) and *wri1* (Focks and Benning, 1998) were also used to validate the method. The *mum4‐1*,* gl2‐1*,* fus3‐3*, and *mum2‐1* alleles used in this study are in the Col‐0 wild‐type background, and *wri1‐1* is in the *Ler* wild‐type background.

As seed oil accumulation in *A. thaliana* is highly dependent on environmental conditions, it is essential that seeds used for density measurements are grown under well‐controlled conditions in the same chamber at the same time. All genotypes described above were grown at 20°C under continuous light (80–150 μmol m^−2^ s^−1^ photosynthetically active radiation [PAR]).

### Separation of high‐ and low‐oil mutants from wild type using CaCl_2_

**Figure 2:**
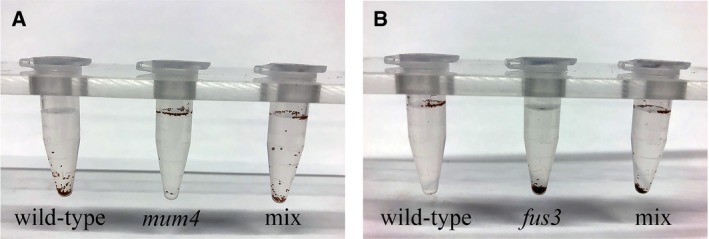
Separation of wild‐type seeds from high‐ and low‐oil mutants. Mature seeds of wild‐type Col‐0 (left), *mum4* (center), and a mixture of these two genotypes (right) were imbibed with 2 M CaCl_2_ and left to settle (A). Wild‐type seeds sink, *mum4* seeds float, and the mixture of both genotypes can be separated. Similarly, mature seeds of wild‐type Col‐0 (left), *fus3* (center), and a mixture of these two genotypes (right) were imbibed with 3 M CaCl_2_ and left to settle (B). Wild‐type seeds float, *fus3* seeds sink, and the mixture of both genotypes can be separated.

Having determined that 30% ethanol was required to prevent mucilage extrusion, a series of solutions of CaCl_2_ in 30% ethanol ranging from 0 M to 3 M in 0.5‐M increments were prepared (seven concentrations in total). For each CaCl_2_ solution tested, approximately 30 seeds from each genotype (Col‐0, *mum4*,* gl2*,* mum2*,* fus3*,* Ler*, and *wri1*) were placed into 1.5‐mL microcentrifuge tubes. One milliliter of each CaCl_2_ solution was added, and the tubes were vortexed briefly to ensure they were wetted before being transferred to a rack for 20–30 min to allow the seeds to settle. Any seeds adhering to the sides of the tube were washed down with extra CaCl_2_ solution. Seeds were visually assessed as to whether they floated or sank. As shown in Table 1, 2 M CaCl_2_ can be used to separate the high‐oil lines *mum4/rhm2* and *gl2* (float) from wild‐type Col‐0 (sink), and 3 M CaCl_2_ can separate the low‐oil lines *fus3* and *wri1* (sink) from wild‐type Col‐0 and *Ler* lines (float), respectively. To illustrate this, we mixed wild‐type and *mum4* seeds and show their separation alongside the individual genotypes using 2 M CaCl_2_ (Fig. 2A). Similarly, the separation of mixed wild‐type and *fus3* seeds alongside the individual genotypes using 3 M CaCl_2_ is shown in Fig. 2B. As expected, *mum2*, which is predicted to have wild‐type oil content, behaved similarly to wild‐type Col‐0. Given that the amount of seed oil accumulated is influenced by the environmental conditions during seed development, the exact concentrations of CaCl_2_ in 30% ethanol required to separate high‐ and low‐density seeds from wild type need to be determined empirically for each experiment performed.

**Table 1:** Separation of seed oil mutants with CaCl_2_ in 30% ethanol.^a^

CaCl_2_ molarity	*gl2*	*mum4/rhm2*	*mum2*	Col‐0	*fus3*	*Ler*	*wri1*
3	f	f	f	f^c^	s^c^	f^c^	s^c^
2.5	f	f	f	f	s	half s, half f	s
2	f^b^	f^b^	most s^b^	most s^b^	s	s	s
1.5	s	s	s	s	s	s	s
1	s	s	s	s	s	s	s
0.5	s	s	s	s	s	s	s
0	s	s	s	s	s	s	s

Note

f = float; s = sink.

a Col‐0 is the wild type for *gl2*,* mum4*,* fus3*, and *mum2*. *Ler* is the wild type for *wri1*.

b At this concentration of CaCl_2_, the high‐oil genotypes *gl2* and *mum4/rhm2* could be separated from wild‐type Col‐0 and the mucilage mutant *mum2*.

c At this concentration of CaCl_2_, the low‐oil genotypes *fus3* and *wri1* could be separated from wild types Col‐0 and *Ler*.

**Table 2:** Screening conditions and seeds recovered from mutagenized populations.

Stock (total seed)^a^	High density/putative low oil			Low density/putative high oil		
	CaCl_2_ concentration, M	Seed recovered (% total seed)	Seed germinated (% seed recovered)	CaCl_2_ concentration, M	Seed recovered (% total seed)	Seed germinated (% seed recovered)
CS21995 (84,000)	4.0	2526 (3.0)	235 (9.3)	1.75	854 (1.0)	729 (85.3)
CS21991 (78,720)	4.5	2700 (3.4)	296 (10.9)	1.75	547 (0.7)	253 (46.3)
CS23153 (62,000)	4.5	874 (1.4)	156 (17.8)	2.0	459 (0.7)	316 (68.8)
CS31100 (930,000)	4.5	13050 (1.4)	775 (5.9)	1.75	1986 (0.2)	1063 (53.5)
RIKEN PSS (293,200)	4.0	3983 (1.4)	467 (11.7)	2.0	1226 (0.4)	796 (64.9)

a The number of seeds screened was calculated based on the number of seeds pooled for each line × the number of lines in each pool × the number of pools in each stock number (Appendix 1).

In order to make sure that seeds were still viable after exposure to ethanol and CaCl_2_, they were rinsed twice in water and then sown onto *Arabidopsis thaliana* (AT) solid growth medium (Haughn and Somerville, 1986) in Petri plates, sealed with micropore tape, and germinated at 20°C under continuous light (80–150 μmol m^−2^ s^−1^ PAR). Control plates consisted of seeds from the same batches planted directly onto plates without exposure to CaCl_2_ or ethanol. After 7 d, the percent germination and growth of germinated seedlings from seeds exposed to density separation solutions were found to be comparable to those that were not exposed, indicating that the density separation solutions did not impact seed germination or seedling health.

### Mutagenized populations screened

Having demonstrated that different concentrations of CaCl_2_ in 30% ethanol can be used to distinguish between genotypes with different oil contents and that seeds remained viable after screening, the method was used to screen three large, publicly available mutagenized populations (Appendix 1). These populations were generated using activation‐tagging vectors that use transcriptional enhancers from the cauliflower mosaic virus (CaMV) 35S promoter. As well as generating knockout mutations by insertion of a transfer DNA (T‐DNA) into the genome, the activation tags also have the potential to generate overexpression phenotypes for genes located adjacent to the T‐DNA (Weigel et al., 2000; Nakazawa et al., 2003). On average, one or two independent T‐DNA insertions are present in the genome of each transformed plant (Alonso et al., 2003), in comparison to approximately 500 point mutations per genome after ethyl methanesulfonate (EMS) mutagenesis (Haughn and Somerville, 1987; Lightner and Caspar, 1998). Therefore, large populations of T‐DNA‐mutagenized plants need to be screened to ensure that the population screened includes mutations in most of the genes of the genome. Given that we screened just over 120,000 T‐DNA insertion lines, and that T‐DNA lines have an average of 1.5 insertions per line (Alonso et al., 2003), there were 180,000 mutations in the populations screened. The *A. thaliana* genome is 135 Mbp, and contains 27,000 genes with an average gene length of 2 kbp (Arabidopsis Genome Initiative, 2000). Therefore, 54 Mbp (40%) of the genome is intragenic, and 72,000 insertions should be located in intragenic sequences. As there are 27,000 genes in *A. thaliana*, each gene should have 2.7 T‐DNA insertions.

### Optimization of screening protocol

Screening for high‐ and low‐oil mutants was carried out sequentially in the same tube (see Appendix 2 for a detailed protocol). Briefly, the high‐concentration CaCl_2_ in 30% ethanol was added to the tube, which was vortexed briefly to ensure all seeds were wetted. Seeds were then left to settle for 20–30 min. Under these conditions, wild‐type seeds were expected to float while high‐density (putative low oil) seeds were expected to sink. If present, the high‐density seeds at the bottom of the tubes were removed, rinsed in water, and transferred to AT plates for germination. The remaining high‐concentration CaCl_2_ solution in the tubes was then removed and replaced with low‐concentration CaCl_2_ in 30% ethanol. Again, the tubes were vortexed briefly to ensure the solution concentration was uniform before the seeds were left to settle for 20–30 min. Wild‐type seeds were expected to sink, while low‐density (putative high oil) seeds were expected to float. Any floating seeds were removed, rinsed in water, and transferred to AT plates for germination. The low‐ and high‐density seeds were germinated as before, and after 7–10 d, seedlings were transferred to prepared soil and grown alongside the appropriate wild‐type controls (20°C, continuous light at 80–150 μmol m^−2^ s^−1^ PAR). Seed from individual plants was harvested and is ready to be rescreened to verify the seed density phenotype by directly measuring the oil content by gas chromatography (Li et al., 2006).

As mentioned above, the concentrations of CaCl_2_ in 30% ethanol must be optimized for seeds grown under different conditions, as even minor changes in growth environment have a large impact on seed oil accumulation. Initial attempts to screen the mutagenized seed pools obtained from the stock centers using the concentrations of CaCl_2_ in 30% ethanol shown in Table 1 (3 M to separate low‐oil mutants from wild type, and 2 M to separate high‐oil mutants from wild type) resulted in the recovery of large numbers of putative seed density mutants. Given that the expected number of mutants with seed oil phenotypes in the population is likely to be relatively small (estimated at less than 50), we assumed that large numbers of false positives were recovered, most likely due to differences in growth conditions at the stock center. Therefore, a small number of vials were tested using different concentrations of CaCl_2_ in 30% ethanol until much smaller numbers of putative mutants were recovered (Table 2). Concomitantly, germination of recovered seeds revealed that not all of the recovered seeds were viable and that the percentage of viable seeds differed between putative low‐oil seeds (6–18% germination, depending on the pool) and putative high‐oil seeds (46–85% germination, depending on the pool). The number of seeds recovered ranged from 1.4–3.4% of the total seeds screened for putative low‐oil mutants and from 0.4–1% for putative high‐oil mutants (Table 2). After screening all vials (approximately 1,447,920 seeds from 120,822 lines in 579 pools; Appendix 1), 3157 putative high‐oil seeds and 1929 low‐oil seeds were regrown. Although this is still a high number of lines, it is possible to rescreen all of these to test for altered oil content using gas chromatography. The stringency of the screen could be increased by adjusting the CaCl_2_ concentration so that fewer putative seed density mutants are recovered.

### Recovery of mutagenized populations for future screening

The mutagenized population can be recovered so that it can be screened again for mutants with any other traits of interest (see Appendix 2 for a detailed protocol). After the low‐density/high‐oil seeds were removed and plated onto AT plates, the screening solution was removed and the remaining seeds (wild‐type density) were rinsed in water. These seeds were transferred into 50 mL of 0.1% w/v agar solution, where they formed a suspension that allows them to be evenly distributed onto prepared soil. The seed from each vial was planted into a separate pot so that seeds corresponding to the original seed pools could be harvested. Alternatively, after rinsing in water as described above, the seed can be dried and stored for future use.

### Identification of new alleles of mutants involved in seed oil accumulation

When seed was harvested from individual plants grown from seeds isolated during the density screen, two lines with interesting phenotypes were visually identified (Appendix S1). One putative high‐oil line (*ttg‐like*) was reminiscent of the *transparent testa glabra1* (*ttg1*; Walker et al., 1999) and *ttg2* mutants (Johnson et al., 2002), with seeds displaying transparent (yellow) testa and no trichomes or seed mucilage, and one putative low‐oil line (*wri1‐like*) was reminiscent of *wri1* with small, dark, wrinkled seeds. A cross to determine genetic complementation between *ttg‐like* and both *ttg1‐1* and *ttg2‐1* revealed that *ttg1‐like* (renamed *ttg1‐2*) is an allele of *ttg1* (At5g24520). *TTG1* is known to be involved in seed oil accumulation in *A. thaliana* (Shi et al., 2012; Chen et al., 2015). Similarly, a cross between *wri1‐like* and *wri1‐1* revealed that *wri‐like* (renamed *wri1‐6*) is an allele of *wri1* (At3g54320). Sequencing of *WRI1* in *wri1‐like* and *TTG1* in *ttg1‐like* confirmed that mutations were present in both genes (Appendix S1). It is interesting to note that neither mutation was associated with a T‐DNA, a phenomenon that has been reported previously (Ajjawi et al., 2010). Segregation analysis was performed by crossing each mutant with Col‐0 wild type and scoring F_2_ progeny for phenotype and genotype. This confirmed that the mutant phenotype(s) segregated with the mutant genotype in both cases. Seed oil content measurements using gas chromatography indicated that *ttg1‐2* has high seed oil content, whereas *wri1‐6* has low seed oil content (Appendix S1).

## CONCLUSIONS

The protocol developed here is a major improvement on previous protocols for separating *A. thaliana* mutants with high and low oil content from wild‐type seeds as it uses nontoxic CaCl_2_ instead of harmful organic chemicals that are difficult to dispose of safely. Furthermore, the addition of ethanol to the CaCl_2_ solution prevents the extrusion of mucilage from *A. thaliana* seeds, which may interfere with the assay. After successfully showing that previously characterized seed oil mutants could be distinguished from wild‐type seeds, the method was used to screen 120,822 T‐DNA mutagenized lines in 579 pools. After this primary screen, 3157 putative high‐oil seeds and 1929 low‐oil seeds were identified and await further analysis. Among these lines were one new allele of *ttg1* (high oil) and one new allele of *wri1* (low oil), demonstrating that the seed density screen can be successfully used to identify novel high‐ and low‐oil mutants.

## AUTHOR CONTRIBUTIONS

G.H.D. designed and carried out experiments, analyzed data, and wrote the manuscript. F.P. designed and carried out experiments and analyzed data. G.W.H. and L.K. designed experiments, analyzed data, and wrote the manuscript. All authors gave final approval to the manuscript before submission and publication.

## Supporting information


**APPENDIX S1.** Characterization of *ttg1‐2* and *wri1‐6*.

## Data Availability

Seed for *ttg1‐2* and *wri1‐6* has been deposited and is publicly available at the Arabidopsis Biological Resource Center (ABRC; https://abrc.osu.edu/).

## Appendix 1 T‐DNA insertion mutant populations screened.

Two populations generated using the activation‐tagging vector pSKI015 developed by Weigel et al. (2000) are available from the Arabidopsis Biological Research Center (ABRC; https://abrc.osu.edu). The Weigel et al. (2000) collection consists of three donations: ABRC stock number CS21995 (84 pools of 100 lines each), CS23153 (62 pools of 100 lines each), and CS21991 (82 pools of 96 lines each) in the Col‐7 background (stock number CS3731). As approximately 10 seeds of each line were used for pooling, each pool contains 1000 seeds. Therefore, the entire Weigel collection consists of 22,472 lines (approximately 224,720 seeds in total) in 228 pools. The Scheible and Somerville collection (Sedbrook et al., 2004; ABRC stock number CS31100) consists of 62,000 lines in 208 pools of 100–350 lines (most have 300–350 lines) in the Col‐2 background (stock number CS907). As 15 seeds per line were used for pooling, the population consists of approximately 930,000 seeds.

The third population was generated in Col‐0 using the pPCVICEn4HPT vector (Hayashi et al., 1992; Nakazawa et al., 2003) and obtained through the RIKEN BioResource Research Center (https://en.brc.riken.jp; RIKEN resource numbers pss00001–pss00009 and pss00101–ps00128). Each resource number listed consists of approximately 20 seed pools containing seed from 50 individual lines (eight seeds per line). To facilitate screening, superpools were generated by mixing five seed pools together, giving a total of 36,650 lines (293,200 seeds) in 143 pools of 250 lines each.

The grand total of lines screened was 120,822 lines in 579 pools (approximately 1,447,920 seeds).

## Appendix 2 Seed density screening protocol.

### Materials and reagents

1. 1.5‐mL microcentrifuge tubes (catalog number: 87003‐294; VWR International, Radnor, Pennsylvania, USA)
2. White paper (only needed if seeds need to be transferred to new tubes for screening)
3. Calcium chloride dihydrate (catalog number: C79‐3; Thermo Fisher Scientific, Waltham, Massachusetts, USA)
4. Distilled water
5. 30% (v/v) ethanol in water
6. Calcium chloride solutions ranging from 0.5–4.5 M in 30% ethanol (see Recipes, below)
7. Plastic Pasteur pipettes, 5.8 mL (catalog number: 612‐4494; VWR International)
8. Plastic rack for 1.5‐mL tubes
9. Porcelain spot plate (e.g., catalog number: J220; Thermo Fisher Scientific)
10. 35‐mm Petri dishes (catalog number: FB0875712; Thermo Fisher Scientific)
11. *Arabidopsis thaliana* (AT) minimal medium, with and without agar (see Recipes)
12. Agar (catalog number: BP1423; Thermo Fisher Scientific)
13. 0.1% (w/v) agar solution (see Recipes; only required for re‐growing populations)
14. Filter paper (only required if populations are to be dried for storage)
15. 1‐L autoclavable bottles for medium preparation
16. Micropore tape (catalog number: 1530‐0; 3M, St. Paul, Minnesota, USA)
17. 1‐mL micropipettor and tips
18. Plant pots, 12 cm diameter
19. Plastic wrap
20. Forceps
21. Sunshine Mix #5 (Sun Gro Horticulture, Agawam, Massachusetts, USA) or growth medium of your choice
22. Plant labels
23. Bamboo barbeque skewers, 30 cm long
24. Aluminum foil
25. Coin envelopes (e.g., Kraft coin envelopes, catalog number: S‐7798; Uline, Pleasant Prairie, Wisconsin, USA)
26. Sealed, airtight boxes for seed storage (e.g., LocknLock 5‐L bread container, catalog number: 094507002TEAL; Starfrit, Longueuil, Québec, Canada)
27. Silica gel (e.g., catalog number: 1019691000; Sigma‐Aldrich Canada Co., Oakville, Ontario, Canada)

### Equipment

1. Vortex mixer with 3‐inch platform (Vortex‐Genie 2, catalog number: SI‐0236; Scientific Industries, Bohemia, New York, USA)
2. Lab balance
3. pH meter
4. Magnetic stirrer and stir bars
5. Autoclave
6. Growth chamber with light level and temperature controls (for plates and growth of plants to maturity)

### Procedure

It is important to keep complete records of which pools were screened and to track the numbers of seeds recovered and germinated from each pool. Seeds that germinate and grow to maturity should be labeled so that it is clear which plants came from which pool. Set up an Excel spreadsheet to enter this data as it is generated.

For each experiment, the concentration of CaCl_2_ in 30% ethanol will need to be optimized to separate seeds with altered density. Adjust the concentrations of high‐ and low‐concentration CaCl_2_ in 30% ethanol to recover appropriate numbers of seeds.

1. If the seeds are already in tubes that are suitable for screening (e.g., microcentrifuge tubes of at least 1.5‐mL volume), there is no need to transfer seeds. If the tubes are not suitable, or if seeds need to be pooled further, then transfer to a 1.5‐mL microcentrifuge tube. If the seeds are hard to remove due to static, flick or knock them out onto a piece of white paper first, then transfer to 1.5‐mL microcentrifuge tubes.
2. Add 1 mL of high‐concentration CaCl_2_ in 30% ethanol to the tube.
3. Vortex briefly (5–10 s) to ensure that all seeds are wetted thoroughly.
4. Using additional screening solution and a plastic Pasteur pipette, wash down any seeds that are stuck to the sides of the tube.
5. Place the tubes into a rack and leave to settle for 20–30 min.
6. Remove sinking seeds (high density/low oil) with a plastic Pasteur pipette.
  - Transfer seeds to a porcelain spot plate and rinse twice in distilled water.
  - Transfer the washed seeds to an AT minimal medium plate.
  - Record the number of seeds recovered and label the plate with the pool number.
  - Seal the plate with micropore tape.
7. Remove the high‐concentration CaCl_2_ using 1‐mL micropipettor.
8. Add 1 mL of low‐concentration CaCl_2_ in 30% ethanol.
9. Vortex briefly (5–10 sec) to ensure that the solution concentration is uniform.
10. Using additional screening solution and a plastic Pasteur pipette, wash down any seeds that are stuck to the sides of the tube.
11. Place the tubes into a rack and leave to settle for 20–30 min.
12. Remove floating seeds (low density/high oil) with a plastic Pasteur pipette.
  - Transfer seeds to a porcelain spot plate and rinse twice in distilled water.
  - Transfer the washed seeds to an AT minimal medium plate.
  - Record the number of seeds recovered, and label the plate with the pool number.
  - Seal the plate with micropore tape.
13. To recover the population, remove the remaining CaCl_2_ in 30% ethanol from the tube using a plastic Pasteur pipette.
14. Wash the remaining seeds in the screening tubes twice in distilled water.
15. Choose one of the following options:
  - Regrow the population immediately. Plant seeds from each screening tube into separate pots to maintain the population's structure.
    - Transfer the washed seeds into 50 mL of 0.1% (w/v) agar solution using a plastic Pasteur pipette. The seeds will suspend in the agar solution.
    - Prepare one pot of growth medium for each tube of seeds and add 30 mL of AT minimal medium without agar to the top of the growth medium.
    - Spread the seeds evenly onto the growth medium using a plastic Pasteur pipette, cover the pots with plastic wrap, and transfer to the growth chamber (20°C under continuous light at 100–150 μmol m^−2^ s^−1^ photosynthetically active radiation [PAR]).
    - Remove plastic wrap after 7–10 d.
    - Grow until seed set is complete, harvest, and store in coin envelopes in airtight boxes with silica gel.
  - Dry the seeds for future use. This needs to be done immediately after screening because if germination is initiated during screening or washing, the seed will die when it is dried.
    - Transfer the washed seeds to filter paper and air dry them, keeping seeds from the same tube separate to maintain the population structure. Dried seeds can be stored in coin envelopes in airtight boxes with silica gel.
16. Transfer the AT minimal medium plates with the seed recovered in steps 6 and 12 to the growth chamber (20°C under continuous light at 100–150 μmol m^−2^ s^−1^ PAR).
17. After 7–10 d, record the number of seeds germinated.
18. Prepare growth medium in pots, and water the top of medium with 30 mL of AT minimal medium without agar.
19. Using forceps, transfer eight seedlings to each pot. Label each plant with the pool from which it was recovered.
20. Cover with plastic wrap, and transfer to the growth chamber (20°C under continuous light at 100–150 μmol m^−2^ s^−1^ PAR).
21. After 3–4 d, remove the plastic wrap.
22. Once the plants have bolted, gather the inflorescence stems of each plant together and tie them to a bamboo skewer using aluminum foil.
23. Water plants until the last siliques are yellow and beginning to dry out to ensure that seed development and oil deposition is complete.
24. Harvest the seeds from individual plants, removing all the chaff. Store seeds in coin envelopes in airtight boxes with silica gel.

### Recipes

#### CaCl_2_ in 30% ethanolUse CaCl_2_·2H_2_O to make solutions as it dissolves more easily than anhydrous CaCl_2_

Prepare a 4.5 M solution by dissolving 330.77 g of CaCl_2_·2H_2_O in 400 mL of 30% (v/v) ethanol. The solution will get hot. After letting it cool, add 30% (v/v) ethanol to a total volume of 500 mL. This solution can then be diluted with 30% (v/v) ethanol to prepare any other concentrations required.

#### AT minimal medium

We use an AT minimal medium that was developed in our lab (Haughn and Somerville, 1986), but the *A. thaliana* growth medium of your choice can be substituted. For AT minimal medium, begin by making the stock solutions (Table A1) and autoclaving them in 1‐L bottles. To prepare the medium, add the correct amount of each nutrient stock solution to 800 mL of distilled water (Table A1). Add stocks to water to prevent precipitation of insoluble calcium phosphate complexes. Add water to a total volume of 1 L.

We water our growth medium with AT minimal medium without agar before planting, but this may not be necessary depending on your choice of growth medium. For watering with AT minimal medium, use directly, but store in the dark to prevent growth of algae.

For plates, adjust the pH of the medium to 5.8 with 1 M KOH, add 7 g/L of agar, and autoclave. After autoclaving, medium can be stored in bottles at room temperature and plates can be stored wrapped in plastic wrap at 4°C.

#### 0.1% (w/v) agar solution

Place 1 g of agar in 1 L of distilled water in a 1‐L autoclavable bottle. Autoclave and allow to cool before use.

**Table A1:** *Arabidopsis thaliana* minimal medium recipe.

Nutrient stock	g/L to prepare stock	mL of stock per 1 L medium
1 M KNO_3_	101.102	5
1 M KH_2_PO_4_	136.1	2.5
1 M MgSO_4_	246.5	2
1 M Ca(NO_3_)_2_	236.2	2
Micronutrients		1
70 mM H_3_BO_3_	4.3	
14 mM MnCl_2_·4H_2_O	2.8	
0.5 mM CuSO_4_	0.29	
1 mM ZnSO_4_·7H_2_O	0.08	
0.2 mM NaMoO_4_·2H_2_O	0.05	
10 mM NaCl	0.58	
0.01 mM CoCl_2_·6H_2_O	0.002	
