# Supplementary material for: A simple, non‐toxic method for separating seeds based on density, and its application in isolating Arabidopsis thaliana seed oil mutants
Source: Appl Plant Sci. 2020 Apr 20;8(4):e11332. doi: 10.1002/aps3.11332 (PMC7186901; doi:10.1002/aps3.11332)
Supplement: Supplementary file 1 — APPENDIX S1. Characterization of ttg1‐2 and wri1‐6. [file APS3-8-e11332-s001.pdf]

APPENDIX S1. Characterization of *ttg1-2* and *wri1-6*.

The *ttg1-like* (*ttg1-2*; yellow seeds) and *wri1-like* (*wri1-6*; small wrinkled seeds) mutants were isolated visually based on their distinctive appearances as shown below (scale bar = 500 μm).

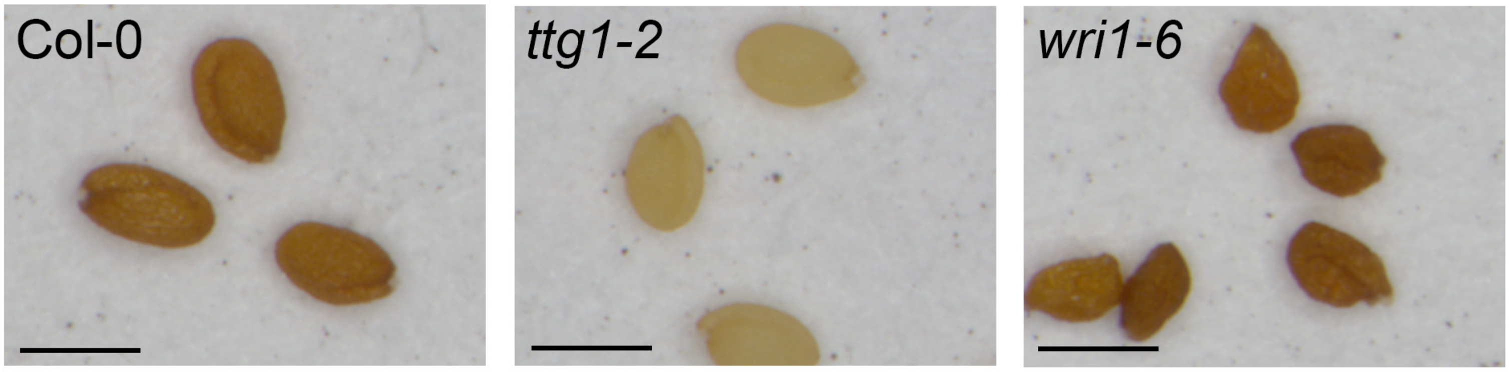

Genomic DNA was extracted from *ttg1-2* and *wri1-6* and the genomic region containing the *TTG1* and *WRI1* genes was amplified from *ttg1-2* and *wri1-6*, respectively. The resulting PCR products were sequenced, and revealed an 11-bp deletion in *ttg1-2*, and the replacement of 31 bp of *wri1-6* with a 5-bp insertion, as depicted in the diagram below.

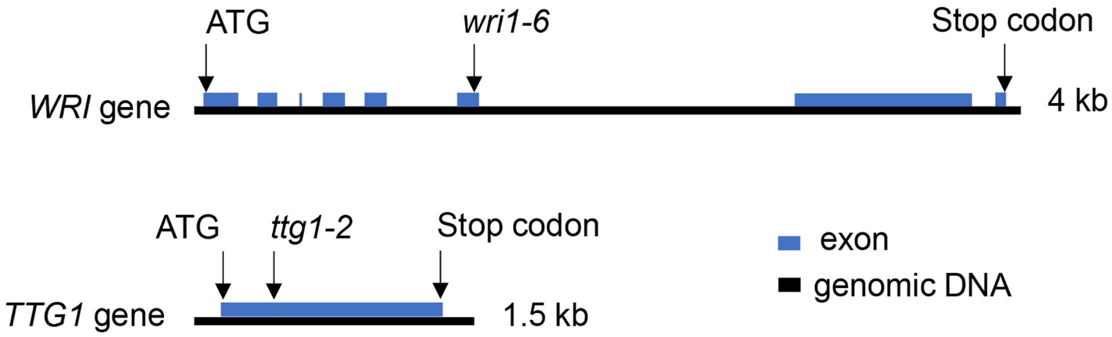

Finally, seed oil content (fatty acid methyl esters, FAMEs) was measured by gas chromatography (Li et al., 2006) and expressed as micrograms of seed oil per milligram of seed (four replicates per genotype, error bars = standard error). The *wri1-1* and *ttg1-1* alleles were included for comparison (*ttg1-1* is in the *Ler* background, *wri1-1* is in the Col-0 background). As shown below, the data indicate that *ttg1-2* has high seed oil content and *wri1-6* has low seed oil content.

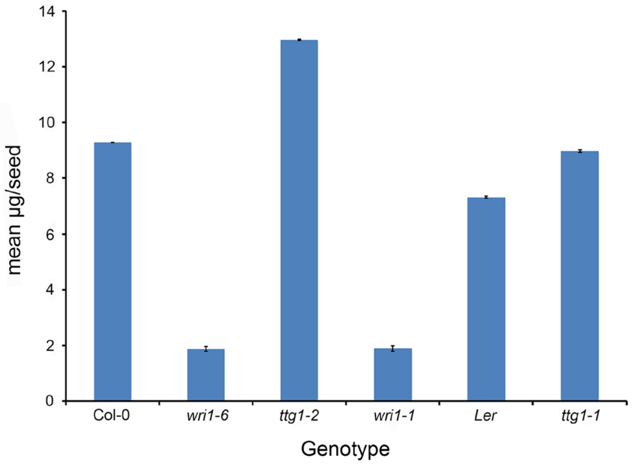

LITERATURE CITED

Li, Y., F. Beisson, M. Pollard, and J. Ohlrogge. 2006. Oil content of Arabidopsis seeds: The influence of seed anatomy, light and plant-to-plant variation. *Phytochemistry* 67: 904–915.
